# Assessment of Chronotype Distribution Among University Students and Its Association With Lifestyle Characteristics and Academic Performance

**DOI:** 10.7759/cureus.67678

**Published:** 2024-08-24

**Authors:** Yara Ajeebi, Imtenan A Oberi, Manal Al-Hulaibi, Bandar A Omair, Ghadi F Alsum, Saliha M Abukhairat, Osama M Abualgasem, Ibrahim M Gosadi

**Affiliations:** 1 College of Medicine, Jazan University, Jazan, SAU; 2 General Surgery, King Fahad General Hospital, Al Ahsa, SAU; 3 Emergency, Jazan General Hospital, Jazan, SAU; 4 Public Health, Jazan University, Jazan, SAU; 5 Family and Community Medicine, Jazan University, Jazan, SAU

**Keywords:** kingdom of saudi arabia (ksa), lifestyle characteristics, academic performance, chronotypes, university students

## Abstract

Background: Chronotype is associated with the timing of peak physical and mental performance and activity levels. University students may experience changes in their chronotype, influencing their daytime activity and academic performance. This study aims to assess the distribution of chronotypes among a sample of university students from southern Saudi Arabia, examining its association with demographic, academic, and lifestyle factors.

Methods: A cross-sectional study was conducted at Jazan University, located in the southwestern region of Saudi Arabia, between February and March 2023. Data collection was carried out using a structured questionnaire comprising three main components: demographic and academic data of participants, lifestyle characteristics, and an assessment of chronotype using the reduced version of the Horne and Östberg morningness-eveningness questionnaire. Associations between chronotype and demographic/lifestyle characteristics were analyzed using Pearson’s chi-squared test or Fisher’s exact test.

Results: The study included 507 students. The mean age of the participants was 22 years (standard deviation: 2.07), with over half being male 277 (54.6%). The chronotype assessment indicated that 139 (27.4%) of students were morning types, 112 (22.1%) evening types, and 256 (50.5%) were neither type. Statistically significant variations in chronotypes were found in relation to the year of study, perceived influence of sleep habits on academic performance, meal frequency, consumption of fast food and certain caffeinated beverages, and smoking or Khat chewing habits (P < 0.05).

Conclusion: The findings suggest that an unhealthy lifestyle and the use of certain stimulants can influence chronotypes. Students with an evening chronotype should be a focus for university health services, allowing early identification and counseling to mitigate the negative impact of a disturbed chronotype on academic performance and reduce the risk of study-related stress.

## Introduction

Sleep quality is a critical component of a healthy lifestyle [1]. Both poor sleep quality and sleep deprivation have been shown to negatively impact health-related quality of life [2,3]. The assessment of sleep quality is recommended as a standard clinical practice, especially among vulnerable groups such as those with chronic diseases [4]. Furthermore, certain populations may be at a higher risk of poor sleep quality depending on their exposure to stressful environments.

Chronotype is a factor that can be associated with sleep quality. It defines an individual’s preference concerning their sleep-wake cycle, classifying individuals as morning, evening, or intermediate types. This classification depends on their usual activity times: morning types are more active in the morning, while evening types are more active in the evening. The type of chronotype is also linked with the timing of peak physical and mental performance [5]. Disturbances in chronotype have been reported to affect mental health [6-8], with evening-type individuals being at a higher risk of mental health problems [9,10]. The vulnerability of certain groups to chronotype disturbances and their impact on mental health has motivated the adoption of better approaches to identify at-risk individuals and the application of suitable therapeutic interventions [7].

University students, often subjected to stressful events, are at risk of poor sleep quality [11]. Students in certain specialties, such as medical students, are reported to be at an even higher risk due to the intensive nature of their education and clinical training [12-14]. Chronotype is associated with sleep quality, where evening types are at a higher risk of poor sleep quality and quantity compared to other types [15].

Studies assessing chronotype among university students have shown varied findings. A study of 759 college students in Saudi Arabia in 2008 found that the majority were neither type (417, 54.9%), followed by evening type (204, 26.9%) [16]. A similar study in Iraqi Kurdistan with 580 medical students in 2013 found that the majority were neither type (305, 52.6%), followed by morning types (141, 24.3%), with no significant gender influence on chronotype [17]. However, a Turkish study with 564 medical students concluded that the majority were neither type (376, 66.7%), followed by 150 were evening types (26.6%), and only 38 (6.7%) were morning types [18], contrasting with similar studies from the Arab world.

Current evidence suggests that chronotype variability can be linked to the variability of individual characteristics. University students may face events that influence their chronotype, which can subsequently affect their daytime activeness and academic performance. This study aims to assess the distribution of chronotypes among a sample of university students from southern Saudi Arabia. Furthermore, it also seeks to assess the demographic and lifestyle factors associated with chronotype distribution.

## Materials and methods

Study context

The current assessment was a cross-sectional investigation conducted between February and March 2023, targeting Jazan University students. Data collection occurred in online settings to reach students affiliated with the university at the time of recruitment. Ethical approval for conducting the study was granted by the Standing Committee of Scientific Research at Jazan University (approval number: REC-44/04/365, dated November 14, 2022). Participation was voluntary, and students had the right to accept participation, reject it, or withdraw at any stage of the study.

Data collection tool

Data was collected using a structured questionnaire, which consisted of three main components. First, the questionnaire gathered demographic data of the participants, including gender, age, marital status, any chronic disease diagnoses, history of smoking and khat chewing, college affiliation, year of study, latest course grade, and GPA (grade point average), and their perceptions of the impact of sleep quality on academic performance. The second part measured lifestyle characteristics associated with sleep quality, adopted from similar literature that provides evidence of factors influencing sleep quality [19-22]. These lifestyle characteristics included the use of mobile phones before sleeping, consumption of meals on weekdays and weekends, consumption of fast food, timing of the last meal, regularity of exercise, timing of tea or coffee consumption, and daily consumption of caffeine-rich food items like tea, coffee, espresso, power drinks, and soft drinks. Finally, the participants’ chronotypes were assessed using the reduced version of the Horne and Östbergmorningness-eveningness questionnaire, with details about the scale’s use and validity described elsewhere. This scale has 19 questions; the scale was developed to assess individual differences in morningness and eveningness - the degree to which respondents are active and alert at certain times of day. The number of points scores can range from 16 to 86. Scores of 41 and below indicate "evening types." Scores of 59 and above indicate "morning types." Scores between 42 and 58 indicate "intermediate types" [23]. The questionnaire was piloted on a sample of 10 male and 10 female students to test the clarity of the questions and the time needed to complete them.

Data collection process

The questionnaire was converted into an online format using Google Forms. A web link was generated for easy access to the questionnaire. Students were identified and approached through advertisements in WhatsApp groups associated with university students. The shared web link included an information letter explaining the study’s aims, the nature of the questionnaire, and the inclusion and exclusion criteria. The inclusion criteria were restricted to current university students. Alumni and non-affiliated individuals were excluded. Participants who consented were given access to the questionnaire, while those who did not consent were directed elsewhere.

Participants in this study were targeted using convenience sampling. The sample size was estimated using the StatCalc function of EpiInfo (Centers for Disease Control and Prevention (CDC), Atlanta, USA) based on the expectation of identifying a 54.9% prevalence of students classified as neither type. This assumption was derived from a previous study in Riyadh, Saudi Arabia, which found that 417 (54.9%) of their students were neither type [16]. An expected frequency of 54.9%, a 95% confidence level, a 5% margin of error, and an anticipated 25% rate of refusal or noninclusion led to a required sample size of 475 university students.

Statistical analysis

Data analysis was conducted using the Statistical Package for the Social Sciences (IBM SPSS Statistics for Windows, IBM Corp., Version 25.0, Armonk, NY). Binary and categorical variables were summarized using frequencies and proportions, while continuous data were summarized using means and standard deviations. The chronotype assessment was performed using the established scoring system described elsewhere [23]. This scale classified students as “morning type,” “neither type,” or “evening type.” The association between chronotype and demographic and lifestyle characteristics was assessed using either Pearson’s chi-squared test or Fisher’s exact test. A P-value of 0.05 or less was considered statistically significant for the applied tests.

## Results

The total number of participants approached in the current investigation was 549. Of these, 42 were excluded due to either not meeting the inclusion criteria or not agreeing to participate. Consequently, the number of individuals involved in the current analysis was 507, which is slightly higher than initially estimated. This increase is attributed to the convenient distribution of the questionnaire web link. The demographic and academic characteristics of the recruited students are displayed in Table 1. The mean age of the participants was 22 years, with a standard deviation of 2.07. Over half of the participants were male (277, 54.6%). The majority of respondents were single (469, 92.5%), not affected by any chronic disease (441, 87%), enrolled in health specialties (338, 66.7%), never smokers (411, 81.7%), and had never chewed Khat (461, 91.7%). The academic characteristics of the students show that most of them were related to health specialties. Half were in their first three years of studies, more than half scored an A+ or A in their last course, and the majority had either excellent or very good GPAs (grade point average). When asked about their perception of the impact of sleep disturbance on academic performance, a significant majority (359, 70.8%) reported that sleep disturbances can have a strong impact on academic performance.

**Table 1:** Demographic and academic characteristics of 507 university students from Jazan, Saudi Arabia. * Note: Four missing cases for smoking history and khat chewing; 14 missing cases for the year of study; 22 missing cases for the latest course grade. GPA: grade point average

Variables*	Frequency (Proportion)
Gender	
Male	277 (54.6%)
Female	230 (45.4%)
Social Status	
Single	469 (92.5%)
Married	34 (6.7%)
Divorced	4 (0.8%)
Diagnosis With a Chronic Disease	
None	441 (87.0%)
Yes	66 (13.0%)
Smoking	
Never	411 (81.7%)
Current	53 (10.5%)
Previous	39 (7.8%)
Khat Chewing	
Never	461 (91.7%)
Current	13 (2.6%)
Previous	29 (5.8%)
College Category	
Health	338 (66.7%)
Science/Art	169 (33.3%)
Year of Study	
1-3 years	250 (50.7%)
4-7 years	243 (49.3%)
Latest Course Grade	
A	260 (53.6%)
B	149 (30.7%)
C or less	76 (15.7%)
GPA	
Excellent	195 (39.65%)
Very good	161 (32.7%)
Good or less	136 (26.8%)
The Belief That Sleep Disturbance Affects Academic Performance	
Strong effect	359 (70.8%)
Moderate effect	130 (25.6%)
No effect	18 (3.6%)

Table 2 displays the lifestyle characteristics associated with sleep behavior among the recruited students. The majority of the students (432, 85.2%) reported ceasing the use of mobile phones less than 30 minutes before going to sleep. More than half of the students (296, 58.4%) consume one or two meals during weekdays, while the proportion of students consuming three or more meals increases during the weekend, suggesting that students consume more meals on weekends. Regarding the consumption of fast food, only 125 (24.7%) indicated that a week would pass without consuming fast food. Questions related to the timing of consuming coffee or tea before sleeping showed that nearly one-fifth of the students consume these products within an hour before going to bed. Inquiries about the consumption of beverages with caffeine content revealed that the most frequently consumed drink is tea, followed by soft drinks and coffee.

**Table 2:** Distribution of lifestyle characteristics associated with sleep behavior among 507 university students from Jazan, Saudi Arabia.

Statement	Frequency (Proportions)
Exercising Regularly at Least Once a Week	
Yes	188 (37%)
No	319 (63%)
Period of Stopping Use of Mobile Phone Before Sleeping	
Less than 30 Minutes	432 (85.2%)
Thirty Minutes or More	75 (14.8%)
Number of Meals Consumed Daily During Weekdays	
One or Two Meals	296 (58.4%)
Three Meals or More	211 (41.6%)
Number of Meals Consumed Daily During Weekends	
One or Two Meals	257 (50.7%)
Three Meals or More	250 (49.3%)
Consumption of a Minimum of One Fast Food Meal Per Week	
Yes	382 (75.3%)
No	125 (24.7%)
Timing of Last Meal Before Going to Bed	
Less than an Hour	191 (37.7%)
An Hour or More	316 (62.3%)
Timing of Last Cup of Coffee or Tea Before Going to Bed	
Less than an Hour	92 (18.1%)
An Hour or More	415 (81.9%)
Daily Consumption of Coffee	
Yes	192 (37.9%)
No	315 (62.1%)
Daily Consumption of Tea	
Yes	235 (46.4%)
No	272 (53.6%)
Daily Consumption of Espresso	
Yes	65 (12.8%)
No	442 (87.2%)
Daily Consumption of Power Drinks	
Yes	76 (15%)
No	431 (85%)
Daily Consumption of Soft Drinks	
Yes	220 (43.4%)
No	287 (56.6%)

The assessment of morningness and eveningness is displayed in Table 3. The most frequently reported preferred waking time was between 5 AM and 6:30 AM, with over half of the respondents indicating that they feel moderately tired upon waking in the morning. The reported preferred time to sleep showed greater variability, with responses ranging from 69 (13.6%) to 140 (27.6%) for various timings. Nonetheless, the most frequently reported bedtime was between 12:30 AM and 3 AM, suggesting that a significant portion of the students go to bed at late hours. When asked about their most active time of the day, over half of the students indicated it was between 8 AM and 5 PM. Finally, when describing themselves as either morning or night people, the most common description leaned toward being a night person (215, 42.4%).

**Table 3:** Morningness and eveningness assessment among 507 university students from Jazan, Saudi Arabia.

Statement	Frequency (Proportions)
Preferred Time to Wake Up	
5 AM to 6:30 AM	220 (43.4%)
6:30 AM to 7:45 AM	99 (19.5%)
7:45 AM to 9:45 AM	95 (18.7%)
9:45 AM to 11 AM	51 (10.1%)
11 AM to 12 PM	42 (8.3%)
Feeling Upon Waking Up Early in the Morning	
Extremely Active	21 (4.1%)
Moderately Active	139 (27.4%)
Moderately Tired	266 (52.5%)
Extremely Tired	81 (16.0%)
Preferred Time to Sleep	
8 PM to 9 PM	72 (14.2%)
9 PM to 10:15 PM	69 (13.6%)
10:15 PM to 12:30 AM	115 (22.7%)
12:30 AM to 1:45 AM	111 (21.9%)
1:45 AM to 3 AM	140 (27.6%)
Time of the Day Which Is Most Active	
5 AM to 8 AM	75 (14.8%)
8 AM to 10 AM	150 (29.6%)
10 AM to 5 PM	122 (24.1%)
5 PM to 10 PM	114 (22.5%)
10 PM to 5 AM	46 (9.1%)
Self-description as Morning or Night Person	
Definitely Morning Person	69 (13.6%)
More Toward Morning Person	146 (28.8%)
More Toward Night Person	215 (42.4%)
Definitely Night Person	77 (15.2%)

The assessment of chronotype, based on the Horne and Östberg morningness-eveningness questionnaire, indicates that 139 (27.4%) of the students are classified as morning types, 112 (22.1%) as evening types, and 256 (50.5%) as neither type. The assessment of the association between demographic, academic, and lifestyle characteristics with students’ chronotypes is displayed in Table 4. This assessment suggests that the history of smoking (0.003), khat chewing (0.022), year of study (0.031), beliefs about the impact of sleep disturbance on academic performance (0.033), period of cessation of mobile phone use before going to bed (0.027), number of meals during weekdays (0.009) or weekends (0.029), and consumption of fast food (0.032), espresso (0.046) or soft drinks daily (0.963) are associated with chronotype with statistically significant differences (P < 0.05). However, no statistically significant variations in chronotypes were identified concerning gender, age, marital status, diagnosis with a chronic disease, academic specialty or performance, timing of the last meal, tea or coffee consumption before bed, daily consumption of coffee, power drinks, tea, or regularity of exercise.

**Table 4:** Assessment of association between demographic, academic, and lifestyle characteristics and chronotype among 507 university students from Jazan, Saudi Arabia. *Pearson’s Chi-squared test, **Fisher’s Exact test; GPA: grade point average

Variables	Chronotype			Total	P-value
	Morning Type	Neither Type	Evening Type		
Gender					
Male	67 (24.2%)	140 (50.5%)	70 (25.3%)	277 (100%)	0.077*
Female	72 (31.3%)	116 (50.4%)	42 (18.3%)	230 (100%)	
Total	139 (27.4%)	256 (50.5%)	112 (22.1%)	507 (100%)	
Age					
Less than 22	58 (24.5%)	132 (55.7%)	47 (19.8%)	237 (100.0%)	0.094*
22 or More	81 (30.0%)	124 (45.9%)	65 (24.1%)	270 (100.0%)	
Total	139 (27.4%)	256 (50.5%)	112 (22.1%)	507 (100.0%)	
Marital Status					
Single	126 (26.9%)	238 (50.7%)	105 (22.4%)	469 (100.0%)	0.617*
Married/Divorced	13 (34.2%)	18 (47.4%)	7 (18.4%)	38 (100.0%)	
Total	139 (27.4%)	256 (50.5%)	112 (22.1%)	507 (100.0%)	
Smoking					
Never	123 (29.9%)	208 (50.6%)	80 (19.5%)	411 (100.0%)	0.003*
Current/Previous	16 (16.7%)	48 (50.0%)	32 (33.3%)	96 (100.0%)	
Total	139 (27.4%)	256 (50.5%)	112 (22.1%)	507 (100.0%)	
Khat Chewing					
Never	133 (28.9%)	232 (50.3%)	96 (20.8%)	461 (100.0%)	0.022*
Current/Previous	6 (13.0%)	24 (52.2%)	16 (34.8%)	46 (100.0%)	
Total	139 (27.4%)	256 (50.5%)	112 (22.1%)	507 (100.0%)	
Year of Study					
1-3 Years	68 (27.2%)	139 (55.6%)	43 (17.2%)	250 (100.0%)	0.031*
4-7 Years	67 (27.6%)	112 (46.1%)	64 (26.3%)	243 (100.0%)	
Total	135 (27.4%)	251 (50.9%)	107 (21.7%)	493 (100.0%)	
The Belief That Sleep Disturbance Can Affect Academic Performance					
Strong Effect	100 (27.9%)	184 (51.3%)	75 (20.9%)	359 (100%)	0.033**
Moderate Effect	32 (24.6%)	69 (53.1%)	29 (22.3%)	130 (100.0%)	
No Effect	7 (38.9%)	3 (16.7%)	8 (44.4%)	18 (100.0%)	
Total	139 (27.4%)	256 (50.5%)	112 (22.1%)	507 (100.0%)	
Latest Grade					
A	73 (28.1%)	135 (51.9%)	52 (20.0%)	260 (100.0%)	0.206*
B	45 (30.2%)	76 (51.0%)	28 (18.8%)	149 (100.0%)	
C or Less	17 (22.4%)	35 (46.1%)	24 (31.6%)	76 (100.0%)	
Total	135 (27.8%)	246 (50.7%)	104 (21.4%)	485 (100.0%)	
Specialty					
Health	96 (28.4%)	166 (49.1%)	76 (22.5%)	338 (100.0%)	0.669*
Science/Art	43 (25.4%)	90 (53.3%)	36 (21.3%)	169 (100.0%)	
Total	139 (27.4%)	256 (50.5%)	112 (22.1%)	507 (100.0%)	
Diagnosis With a Chronic Disease					
None	127 (28.8%)	221 (50.1%)	93 (21.1%)	441 (100.0%)	0.140*
Yes	12 (18.2%)	35 (53.0%)	19 (28.8%)	66 (100.0%)	
Total	139 (27.4%)	256 (50.5%)	112 (22.1%)	507 (100.0%)	
GPA					
Excellent	58 (29.7%)	94 (48.2%)	43 (22.1%)	195 (100.0%)	0.610*
Very Good	49 (30.4%)	79 (49.1%)	33 (20.5%)	161 (100.0%)	
Good or Less	31 (22.8%)	73 (53.7%)	32 (23.5%)	136 (100.0%)	
Total	138 (28.0%)	246 (50.0%)	108 (22.0%)	492 (100.0%)	
Last Use of Mobile Phone Before Sleep					
Less than 30 Minutes	110 (25.5%)	228 (52.8%)	94 (21.8%)	432 (100.0%)	0.027*
30 Minutes or More	29 (38.7%)	28 (37.3%)	18 (24.0%)	75 (100.0%)	
Total	139 (27.4%)	256 (50.5%)	112 (22.1%)	507 (100.0%)	
Meals Consumption During Weekdays					
1 or 2	80 (27.0%)	137 (46.3%)	79 (26.7%)	296 (100.0%)	0.009*
Three or More	59 (28.0%)	119 (56.4%)	33 (15.6%)	211 (100.0%)	
Total	139 (27.4%)	256 (50.5%)	112 (22.1%)	507 (100.0%)	
Meals Consumption During the Weekends					
1 or 2	61 (23.7%)	128 (49.8%)	68 (26.5%)	257 (100.0%)	0.029*
Three or More	78 (31.2%)	128 (51.2%)	44 (17.6%)	250 (100.0%)	
Total	139 (27.4%)	256 (50.5%)	112 (22.1%)	507 (100.0%)	
Fast Food Consumption During a Week					
No	45 (36.0%)	59 (47.2%)	21 (16.8%)	125 (100.0%)	0.032*
Yes	94 (24.6%)	197 (51.6%)	91 (23.8%)	382 (100.0%)	
Total	139 (27.4%)	256 (50.5%)	112 (22.1%)	507 (100.0%)	
Timing of Last Meal Before Going to Bed					
Less Than an Hour	52 (27.2%)	94 (49.2%)	45 (23.6%)	191 (100.0%)	0.827*
An Hour or More	87 (27.5%)	162 (51.3%)	67 (21.2%)	316 (100.0%)	
Total	139 (27.4%)	256 (50.5%)	112 (22.1%)	507 (100.0%)	
Timing of Last Tea or Coffee Before Going to Bed					
Less Than an Hour	31 (33.7%)	37 (40.2%)	24 (26.1%)	92 (100.0%)	0.092*
An Hour or More	108 (26.0%)	219 (52.8%)	88 (21.2%)	415 (100.0%)	
Total	139 (27.4%)	256 (50.5%)	112 (22.1%)	507 (100.0%)	
Daily Consumption of Coffee					
Yes	53 (27.6%)	98 (51.0%)	41 (21.4%)	192 (100.0%)	0.963*
No	86 (27.3%)	158 (50.2%)	71 (22.5%)	315 (100.0%)	
Total	139 (27.4%)	256 (50.5%)	112 (22.1%)	507 (100.0%)	
Daily Consumption of Espresso					
Yes	26 (40.0%)	26 (40.0%)	13 (20.0%)	65 (100.0%)	0.046*
No	113 (25.6%)	230 (52.0%)	99 (22.4%)	442 (100.0%)	
Total	139 (27.4%)	256 (50.5%)	112 (22.1%)	507 (100.0%)	
Daily Consumption of Power Drink					
Yes	24 (31.6%)	36 (47.4%)	16 (21.1%)	76 (100.0%)	0.686*
No	115 (26.7%)	220 (51.0%)	96 (22.3%)	431 (100.0%)	
Total	139 (27.4%)	256 (50.5%)	112 (22.1%)	507 (100.0%)	
Daily Consumption of Tea					
Yes	58 (24.7%)	124 (52.8%)	53 (22.6%)	235 (100.0%)	0.447*
No	81 (29.8%)	132 (48.5%)	59 (21.7%)	272 (100.0%)	
Total	139 (27.4%)	256 (50.5%)	112 (22.1%)	507 (100.0%)	
Daily Consumption of Soft Drinks					
Yes	56 (25.5%)	104 (47.3%)	60 (27.3%)	220 (100.0%)	0.049*
No	83 (28.9%)	152 (53.0%)	52 (18.1%)	287 (100.0%)	
Total	139 (27.4%)	256 (50.5%)	112 (22.1%)	507 (100.0%)	
Exercising Regularly at Least Once a Week					
Yes	57 (30.3%)	91 (48.4%)	40 (21.3%)	188 (100.0%)	0.538*
No	82 (25.7%)	165 (51.7%)	72 (22.6%)	319 (100.0%)	
Total	139 (27.4%)	256 (50.5%)	112 (22.1%)	507 (100.0%)	

## Discussion

This study was a cross-sectional investigation aimed at assessing the chronotypes of university students in Saudi Arabia and examining the variation in chronotypes according to demographic, lifestyle, and academic characteristics. The findings indicate that the majority of the students can be classified as neither type. Factors associated with chronotypes suggest that smokers and khat chewers are more likely to be night types. Additionally, a higher proportion of students in their final years are labeled as night persons compared to those in earlier years of study. Students who believe that sleep disturbance has no effect on academic performance were more often classified as night persons. A higher proportion of students who reported ceasing phone use more than 30 minutes before bed were classified as morning types. Students who consume more meals tend to be classified as either morning type or neither type. Those who reported not consuming fast food on a weekly basis were also more likely to be classified as either morning type or neither type. Finally, students who reported daily consumption of espresso were predominantly morning type or neither type, while those consuming tea daily were more often night type or neither type.

The findings of this study can be compared to similar national and international studies. In a 2008 study by BaHammam et al., which involved 759 students from a Saudi university, the most commonly reported chronotype was neither type (417, 54.9%) [16], aligning with the findings of the current study. However, BaHammam et al. found a higher frequency of evening types compared to morning types, contrasting with the current study, where morning types were more frequent. Furthermore, BaHammam et al. investigated the influence of gender on chronotype variation and found no statistically significant difference, a finding similar to that of the current study.

The current study identified statistically significant variations in chronotypes associated with specific dietary habits, such as meal frequency, fast food consumption, and the intake of certain caffeinated beverages. These findings are comparable to a study by Al-Hazmi and Noorwali, which recruited 599 adults from Saudi Arabia aged 18 to 50 years to assess the influence of eating behavior on the chronotypes of their sample. Al-Hazmi and Noorwali reported that the majority of their sample was either morning type or neither type, which parallels our findings. Additionally, they found that evening types were more likely to find fast foods, such as fried foods and fries, more appealing than morning types [24]. This aligns with our findings, where a higher frequency of fast food consumers were classified as evening types.

Comparing our study’s results to similar regional and international investigations revealed comparable findings. A study involving 580 students from Iraq [17] noted that the majority of the students were either “neither type” or “morning type,” akin to our findings. However, a gender variation according to chronotype was detected in the Iraqi study, where females were more likely to be evening types compared to male students, differing from our study, where no statistical variation according to gender was detected.

In another study from Turkey involving 564 medical students aged 17 to 26 years, the majority were either neither type nor evening type, with morning types representing a minority (38, 6.7%) [18]. The findings of the Turkish study significantly differ from those of the current study and other studies conducted in Saudi Arabia, where morning types were relatively higher. This may indicate variations in sleeping habits across different cultures. Additionally, religious and cultural factors, such as changes in sleeping habits during Ramadan among Muslims, where fasting during the daytime was reported to increase daytime sleepiness [21], may affect chronotypes.

The current study did not find a statistically significant variation in chronotypes concerning GPA, latest course grade, or type of specialty. However, the year of study was associated with a statistically significant difference, with a higher frequency of night chronotypes in advanced years compared to those in earlier study years. Additionally, those who do not perceive sleeping habits as having an influence on academic performance were more likely to be evening types. It is important to note that studies assessing the influence of chronotype on academic performance are limited. However, similar studies have included chronotype in the assessment of other variables affecting academic performance. For instance, a 2017 Saudi study involving 169 medical students found no statistically significant correlations between meal consumption, GPA, and chronotype [25]. Moreover, a Mexican study involving 1,086 medical students concluded that perceived academic stress and being an evening type were associated with higher odds of depression [26].

The current study suggests that the frequency of individuals who are smokers or khat chewers is higher among the night type compared to other types, implying that an unhealthy lifestyle can influence chronotype. This finding is supported by a large-scale US study involving 63,676 nurses, which found that those with unhealthy lifestyle choices were more likely to be evening types [27]. Additionally, a report based on the findings of the UK Biobank, with a sample of 439,933, indicated that tobacco product consumption was associated with a higher likelihood of being late chronotypes [28].

The findings of the current study indicate that the use of khat, espresso, and tea is associated with the distribution of chronotypes to varying degrees. This suggests that stimulants might influence chronotypes. However, the influence of stimulants on chronotype has been reported to vary according to age groups and the time of stimulant consumption [29], which was not assessed in this study. This omission suggests the need for further follow-up investigations. Nonetheless, studies assessing the influence of khat chewing in African populations have indicated that khat chewing, which has stimulant properties, is associated with poor sleep quality [30,31]. However, evidence concerning the influence of khat chewing on chronotype is limited.

This study has multiple strengths and weaknesses. The main strength of the study is its ability to reach a sample of university students with a variety of academic, demographic, and lifestyle characteristics and the ability to assess chronotype variations according to these determinants. A weakness of the study is its reliance on convenient sampling and a brief assessment of certain lifestyle factors. However, this study represents preliminary findings that should be followed up by more detailed retrospective or prospective investigations.

## Conclusions

The findings of this study indicate that the majority of students at Jazan University are categorized as neither or morning chronotypes. The year of study, perceptions about the influence of sleep quality on academic performance, and various lifestyle determinants were associated with the distribution of chronotype. Some unhealthy lifestyle practices were linked to a higher likelihood of being an evening type. These findings underscore the importance of conducting more detailed, large-scale investigations to assess how specific lifestyle determinants interact to influence chronotype. Finally, practical implications can be derived from these findings, suggesting that students who are evening types should be targeted by university health services. This would allow for early identification and counseling to mitigate the negative impact of a disturbed chronotype on academic performance and reduce the risk of stress associated with studying.

## References

[1] (2024). American College of Lifestyle Medicine. Medicine.

[2] Lo CMH, Lee PH (2012). Prevalence and impacts of poor sleep on quality of life and associated factors of good sleepers in a sample of older Chinese adults. Health Qual Life Outcomes.

[3] Jemilohun AC, Fasesan OA, Ajiro TO, Akande KO, Elikwu CJ, Adeleye OO (2022). Sleep quality in a Nigerian community: prevalence of poor sleep quality, risk factors and health-related quality of life. West Afr J Med.

[4] Bani-Issa W, Al-Shujairi AM, Patrick L (2018). Association between quality of sleep and health-related quality of life in persons with diabetes mellitus type 2. J Clin Nurs.

[5] Zou H, Zhou H, Yan R, Yao Z, Lu Q (2022). Chronotype, circadian rhythm, and psychiatric disorders: recent evidence and potential mechanisms. Front Neurosci.

[6] Müller MJ, Cabanel N, Olschinski C, Jochim D, Kundermann B (2015). Chronotypes in patients with nonseasonal depressive disorder: distribution, stability and association with clinical variables. Chronobiol Int.

[7] Taylor BJ, Hasler BP (2018). Chronotype and mental health: recent advances. Curr Psychiatry Rep.

[8] Melo MC, Abreu RL, Linhares Neto VB, de Bruin PF, de Bruin VM (2017). Chronotype and circadian rhythm in bipolar disorder: a systematic review. Sleep Med Rev.

[9] Simor P, Zavecz Z, Pálosi V, Török C, Köteles F (2015). The influence of sleep complaints on the association between chronotype and negative emotionality in young adults. Chronobiol Int.

[10] Au J, Reece J (2017). The relationship between chronotype and depressive symptoms: a meta-analysis. J Affect Disord.

[11] Lemma S, Gelaye B, Berhane Y, Worku A, Williams MA (2012). Sleep quality and its psychological correlates among university students in Ethiopia: a cross-sectional study. BMC Psychiatry.

[12] Alotaibi AD, Alosaimi FM, Alajlan AA, Bin Abdulrahman KA (2020). The relationship between sleep quality, stress, and academic performance among medical students. J Family Community Med.

[13] Nsengimana A, Mugabo E, Niyonsenga J (2023). Sleep quality among undergraduate medical students in Rwanda: a comparative study. Sci Rep.

[14] Toubasi AA, Khraisat BR, AbuAnzeh RB, Kalbouneh HM (2022). A cross sectional study: The association between sleeping quality and stress among second and third medical students at the University of Jordan. Int J Psychiatry Med.

[15] Vitale JA, Roveda E, Montaruli A, Galasso L, Weydahl A, Caumo A, Carandente F (2015). Chronotype influences activity circadian rhythm and sleep: differences in sleep quality between weekdays and weekend. Chronobiol Int.

[16] BaHammam AS, Almistehi W, Albatli A, AlShaya S (2011). Distribution of chronotypes in a large sample of young adult Saudis. Ann Saudi Med.

[17] Raoof AM, Asaad YA, Al-Hadithi TS (2014). Distribution of chronotypes among a sample of Iraqi Kurdish medical students. Sultan Qaboos Univ Med J.

[18] Tan MN, Mevsim V, Pozlu Cifci M (2020). Who is happier among preclinical medical students: the impact of chronotype preference. Chronobiol Int.

[19] HHS. Physical Activity Guidelines for Americans 2 nd edition (2018). Physical activity guidelines for Americans 2nd edition. https://health.gov/sites/default/files/2019-09/Physical_Activity_Guidelines_2nd_edition.pdf.

[20] Frimpong E, Mograss M, Zvionow T, Dang-Vu TT (2021). The effects of evening high-intensity exercise on sleep in healthy adults: a systematic review and meta-analysis. Sleep Med Rev.

[21] Alzhrani A, Alhussain MH, BaHammam AS (2022). Changes in dietary intake, chronotype and sleep pattern upon Ramadan among healthy adults in Jeddah, Saudi Arabia: a prospective study. Front Nutr.

[22] Whittier A, Sanchez S, Castañeda B, Sanchez E, Gelaye B, Yanez D, Williams MA (2014). Eveningness chronotype, daytime sleepiness, caffeine consumption, and use of other stimulants among Peruvian University students. J Caffeine Res.

[23] Adan A, Almirall H (1991). Horne & Östberg morningness-eveningness questionnaire: a reduced scale. Pers Individ Dif.

[24] Al-Hazmi MH, Noorwali EA (2023). Morning individuals in Saudi Arabia have higher self-regulation of eating behavior compared to evening types. Chronobiol Int.

[25] Mirghani HO, Albalawi KS, Alali OY, Albalawi WM, Albalawi KM, Aljohani TR, Albalawi WS (2019). Breakfast skipping, late dinner intake and chronotype (eveningness-morningness) among medical students in Tabuk City, Saudi Arabia. Pan Afr Med J.

[26] Romo-Nava F, Tafoya SA, Gutiérrez-Soriano J (2016). The association between chronotype and perceived academic stress to depression in medical students. Chronobiol Int.

[27] Kianersi S, Liu Y, Guasch-Ferré M, Redline S, Schernhammer E, Sun Q, Huang T (2023). Chronotype, unhealthy lifestyle, and diabetes risk in middle-aged U.S. women: a prospective cohort study. Ann Intern Med.

[28] Patterson F, Malone SK, Lozano A, Grandner MA, Hanlon AL (2016). Smoking, screen-based sedentary behavior, and diet associated with habitual sleep duration and chronotype: data from the UK Biobank. Ann Behav Med.

[29] Siudej K, Malinowska-Borowska J (2021). Relationship between chronotype and consumption of stimulants. Chronobiol Int.

[30] Berhanu H, Mossie A, Tadesse S, Geleta D (2018). Prevalence and associated factors of sleep quality among adults in Jimma town, southwest Ethiopia: a community-based cross-sectional study. Sleep Disord.

[31] Jemere T, Mossie A, Berhanu H, Yeshaw Y (2019). Poor sleep quality and its predictors among type 2 diabetes mellitus patients attending Jimma University Medical Center, Jimma, Ethiopia. BMC Res Notes.

